# Re-evaluation of serum leptin and adiponectin concentrations normalized by body fat mass in patients with rheumatoid arthritis

**DOI:** 10.1038/s41598-020-73068-2

**Published:** 2020-09-28

**Authors:** Kazuhisa Chihara, Naoki Hattori, Norihiro Ichikawa, Takeshi Matsuda, Takanori Saito

**Affiliations:** 1 Department of Orthopedics, Kansai Medical University, Osaka, Japan; 2 Department of Pharmaceutical Sciences, Ritsumeikan University, 1-1-1 Nojihigashi, Kusatsu-city, Shiga 525-8577 Japan

**Keywords:** Rheumatic diseases, Obesity

## Abstract

Leptin and adiponectin are produced mainly in adipocytes and classified as adipocytokines because of their possible involvement in inflammation and immunity. The aim of this study was to elucidate the relationships of these adipocytokines with the disease activities of RA. We examined leptin and adiponectin concentrations and inflammatory markers such as metalloproteinase-3 (MMP-3) in 136 patients with rheumatoid arthritis (RA) (26 males and 110 females, 69.6 ± 9.3 years) and 78 controls (36 males and 42 females, 66.7 ± 15.0 years). Serum leptin and adiponectin concentrations correlated positively (r = 0.565, *P* < 0.001) and negatively (r = –0.331, *P* < 0.001) to the amount of body fat, respectively. Serum leptin and adiponectin concentrations normalized by body fat mass were significantly higher in RA than those in controls [leptin, 1.24 (median) ng/mL/kg fat in RA vs. 0.76 ng/mL/kg fat in controls; adiponectin, 0.74 μg/mL/kg fat in RA vs. 0.44 μg/mL/kg fat in controls]. Normalized adiponectin concentrations correlated positively not only to the degree of bone destruction in Steinbrocker classification but also to serum MMP-3 concentrations. Normalized leptin concentrations did not correlate to the degree of bone destruction. We conclude that adiponectin but not leptin may be involved in joint damage in RA.

## Introduction

Adipocytes produce several types of cytokines, termed adipocytokines, which include leptin and adiponectin^1,2^. Leptin binds to the hypothalamic leptin receptor (Ob-R), leading to enhanced metabolism and reduced appetite. Adiponectin binds to adiponectin receptors (adipoR1 and adipoR2) and enhances insulin sensitivity through increased fatty acid oxidation and inhibition of hepatic glucose production. Both leptin and adiponectin are also involved in inflammatory processes and the immune system^3,4^. Leptin increases the release of pro-inflammatory cytokines, such as tumor necrosis factor (TNF)-α, interleukin (IL)-6, and IL-12, from mouse macrophages and activates human blood neutrophils. Conversely, adiponectin has been reported to induce the production of anti-inflammatory cytokines, such as IL-10 and IL-1 receptor antagonist, in a human leukemia cell line and suppress the release of pro-inflammatory cytokines, such as TNF-α from porcine macrophages. Serum leptin concentrations are increased in patients with obesity, type 2 diabetes, metabolic syndrome, and cardiovascular disease, whereas serum adiponectin concentrations are decreased in these disorders. In contrast, high serum levels of adiponectin are reported in autoimmune disorders such as type 1 diabetes, rheumatoid arthritis (RA), systemic lupus erythematosus (SLE), and inflammatory bowel disease^5^.

RA is a chronic systemic autoimmune disease characterized by synovitis^6^. The synovial inflammation leads to cartilage destruction, bone erosion, and subsequent joint deformity. Cytokines such as TNF-α and IL-6 are over-produced by macrophages in the synovial compartment and mediate the inflammatory reactions. Biological disease-modifying anti-rheumatic drugs (DMARDs) targeting these cytokines have improved the prognosis of patients with RA. Serum leptin and adiponectin concentrations have been investigated to examine their potential role in the pathogenesis of RA and to identify possible therapeutic targets^7,8^. It is widely accepted that serum levels of both leptin and adiponectin are elevated in RA^9–12^. However, the relationships of these adipocytokines to the disease activities of RA, particularly the progression of joint damage, are controversial^5,7,10,11,13^. The factor that hampers such analyses might be the great influence of body fat mass because both of these adipocytokines are produced mainly by adipocytes. Serum concentration of leptin increases as body fat mass increases^1^, whereas serum adiponectin concentration paradoxically decreases as body fat mass increases probably because of the suppressive effect TNF-α on adiponectin production^2,14^. Body mass index (BMI), body weight normalized by height squared (kg/m^2^), is a very simple and inexpensive method to estimate body fat. However, for a given BMI, the body fat percentage changes with age, sex, ethnicity and other individual traits^15^.

In the present study, we measured the amount of body fat using a body composition analyzer and normalized serum concentrations of leptin and adiponectin by the amount of body fat. We analyzed serum concentrations of leptin and adiponectin concentrations expressed by non-normalized data, those normalized by BMI and those normalized by the amount of body fat, and re-evaluated their relationships with disease activities of RA, particularly the progression of joint damage.

## Results

### Correlation of serum leptin and adiponectin concentrations with body fat mass

**Figure 1:**
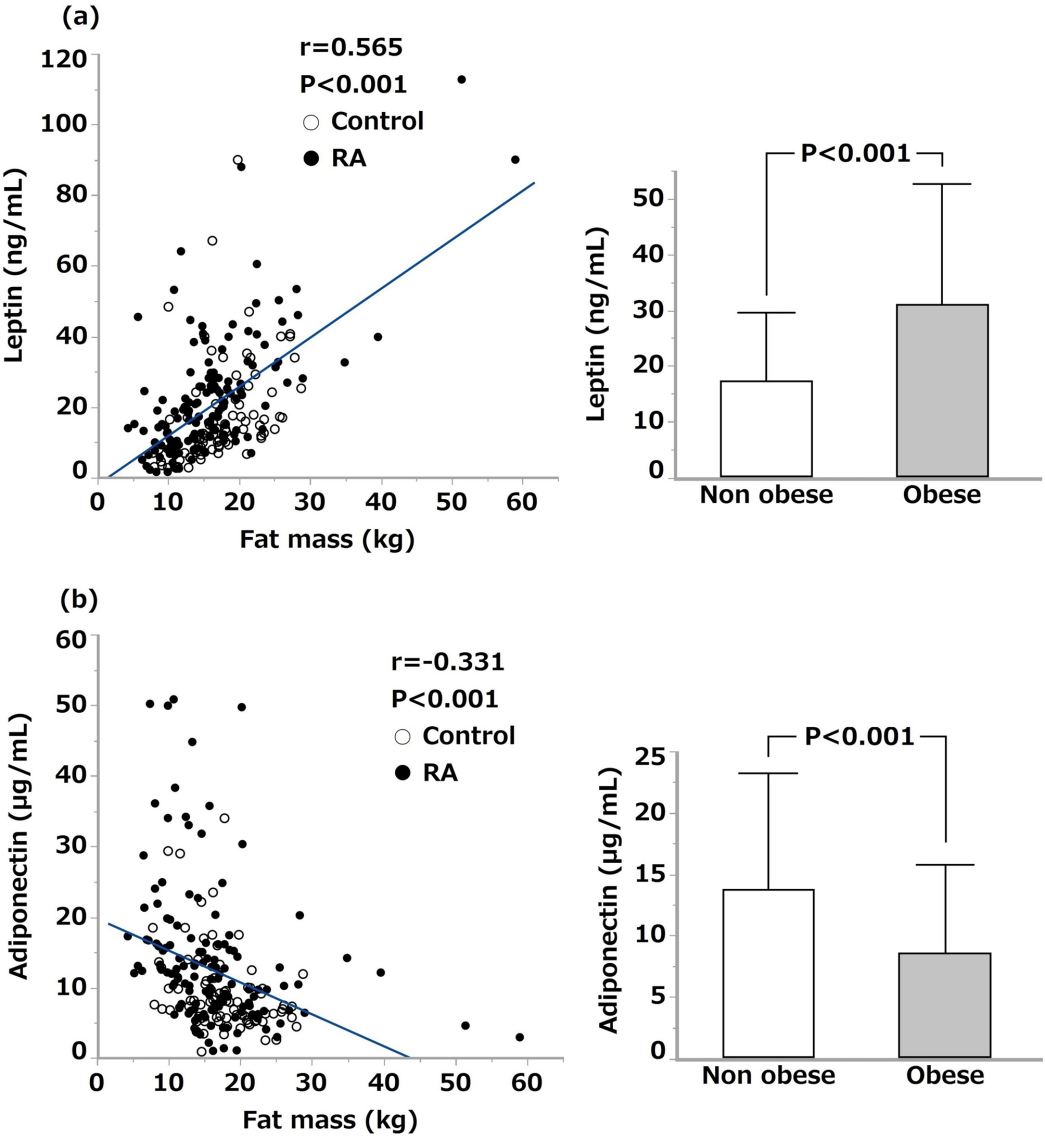
Correlation of serum leptin and adiponectin concentrations with body fat mass. (**a**) shows the relationship between serum leptin and body fat mass in 136 patients with RA (filled circle) and 78 controls (white circle). The right figure demonstrates the comparison of serum leptin concentrations (mean ± SD) between obese (BMI ≥ 25, n = 59) and non-obese (BMI < 25, n = 155) subjects. (**b**) shows the relationship between serum adiponectin and body fat mass in 136 patients with RA (filled circle) and 78 controls (white circle). The right figure demonstrates the comparison of serum adiponectin concentrations between obese and non-obese subjects. Pearson’s correlation coefficient and student’s t test were used for the statistical analyses. *P* < 0.05 was considered statistically significant.

Serum leptin concentration was positively correlated with body fat mass in patients with RA (n = 136, r = 0.661, *P* < 0.001), as well as in controls (n = 78, r = 0.380, *P* < 0.001). Likewise, serum leptin concentration was positively correlated with body fat mass in the total study population (n = 214), as shown in Fig. 1a (r = 0.565, *P* < 0.001). BMI greater than 25 is the criteria for the diagnosis of obesity in Japan^16^. Serum leptin concentration in obese subjects (31.0 ± 21.8 ng/mL, n = 59) was significantly higher than that in non-obese subjects (17.2 ± 12.4 ng/mL, n = 155). Alternatively, serum adiponectin concentration was negatively correlated with body fat mass in patients with RA (r = – 0.312, *P* < 0.001), as well as in controls (r = – 0.334, *P* = 0.003). Serum adiponectin concentration was likewise negatively correlated with body fat mass in the total study population, as shown in Fig. 1b (r = – 0.331, P < 0.001). Serum adiponectin concentration in obese subjects (8.6 ± 7.3 µg/mL) was significantly lower than that in non-obese subjects (13.7 ± 9.6 µg/mL). There was no significant correlation between serum leptin and adiponectin concentrations (r = – 0.121, *P* = 0.078).

### Comparison of serum leptin and adiponectin concentrations between patients with RA and controls

**Figure 2:**
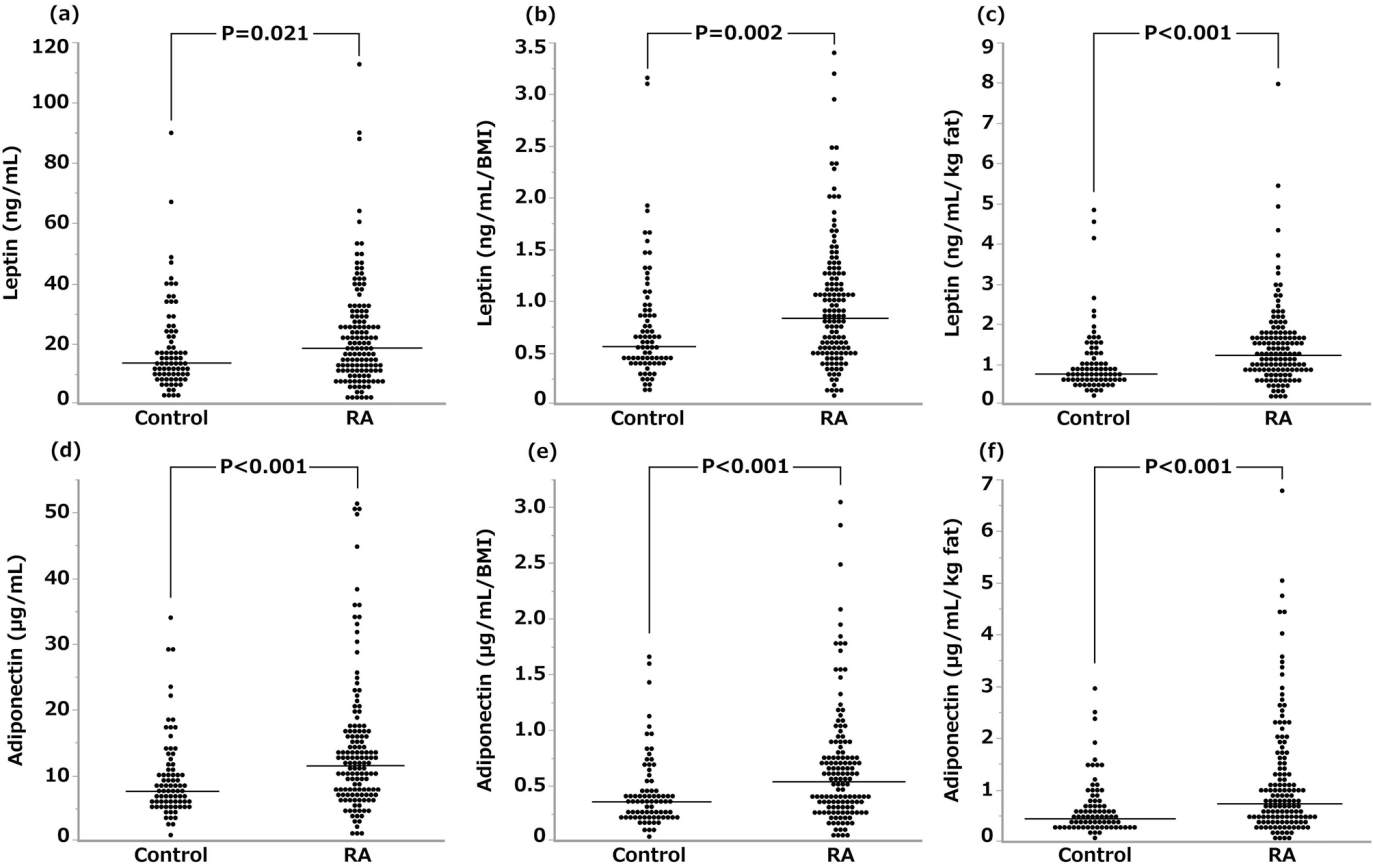
Comparison of serum leptin and adiponectin concentrations between patients with RA and controls. Serum leptin concentrations expressed by raw data (**a**), those corrected by BMI (**b**) and those corrected by body fat mass (**c**); and serum adiponectin concentrations expressed by raw data (**d**), those corrected by BMI (**e**) and those corrected by body fat mass (**f**) in 136 patients with RA and 78 controls are shown. The horizontal line indicates the median value in each group. Wilcoxon rank sum test was used for the statistical analyses. *P* < 0.05 was considered statistically significant.

Figure 2 shows serum leptin and adiponectin concentrations expressed by non-normalized data (raw data), those normalized by BMI (raw data divided by BMI) and those normalized by body fat mass [raw data divided by the amount of body fat (kg)] in patients with RA and controls. Serum leptin concentrations expressed in three ways were all significantly higher in patients with RA than those in controls [median 18.9 ng/mL (interquartile range: IQR 10.9–28.2 ng/mL) vs. 13.8 ng/mL (8.7–23.3 ng/mL), *P* = 0.021; 0.84 ng/mL/BMI (0.49–1.25 ng/mL/BMI) vs. 0.56 ng/mL/BMI (0.40–0.90 ng/mL/BMI), *P* = 0.002; 1.24 ng/mL/kg fat (0.82–1.72 ng/mL/kg fat) vs. 0.76 ng/mL/kg fat (0.54–1.23 ng/mL/kg fat), *P* < 0.001]. However, dispersion of the data of serum leptin concentrations tended to become smaller when they were normalized by body fat mass than that of non-normalized data and normalized ones by BMI, and the statistical difference between patients with RA and controls became more significant. Likewise, serum adiponectin concentrations expressed by three ways were all significantly higher in patients with RA than those in controls [11.5 µg/mL (7.0–16.2 µg/mL) vs. 7.7 µg/mL (5.7–11.1 µg/mL), *P* < 0.001; 0.54 µg/mL/BMI (0.31–0.79 µg/mL/BMI) vs. 0.36 µg/mL/BMI (0.22–0.47 µg/mL/BMI), *P* < 0.001; 0.74 µg/mL/kg fat (0.41–1.50 µg/mL/kg fat) vs. 0.44 µg/mL/kg fat (0.27–0.77 µg/mL/kg fat), *P* < 0.001].

### Correlation of serum leptin and adiponectin concentrations with inflammatory markers

**Figure 3:**
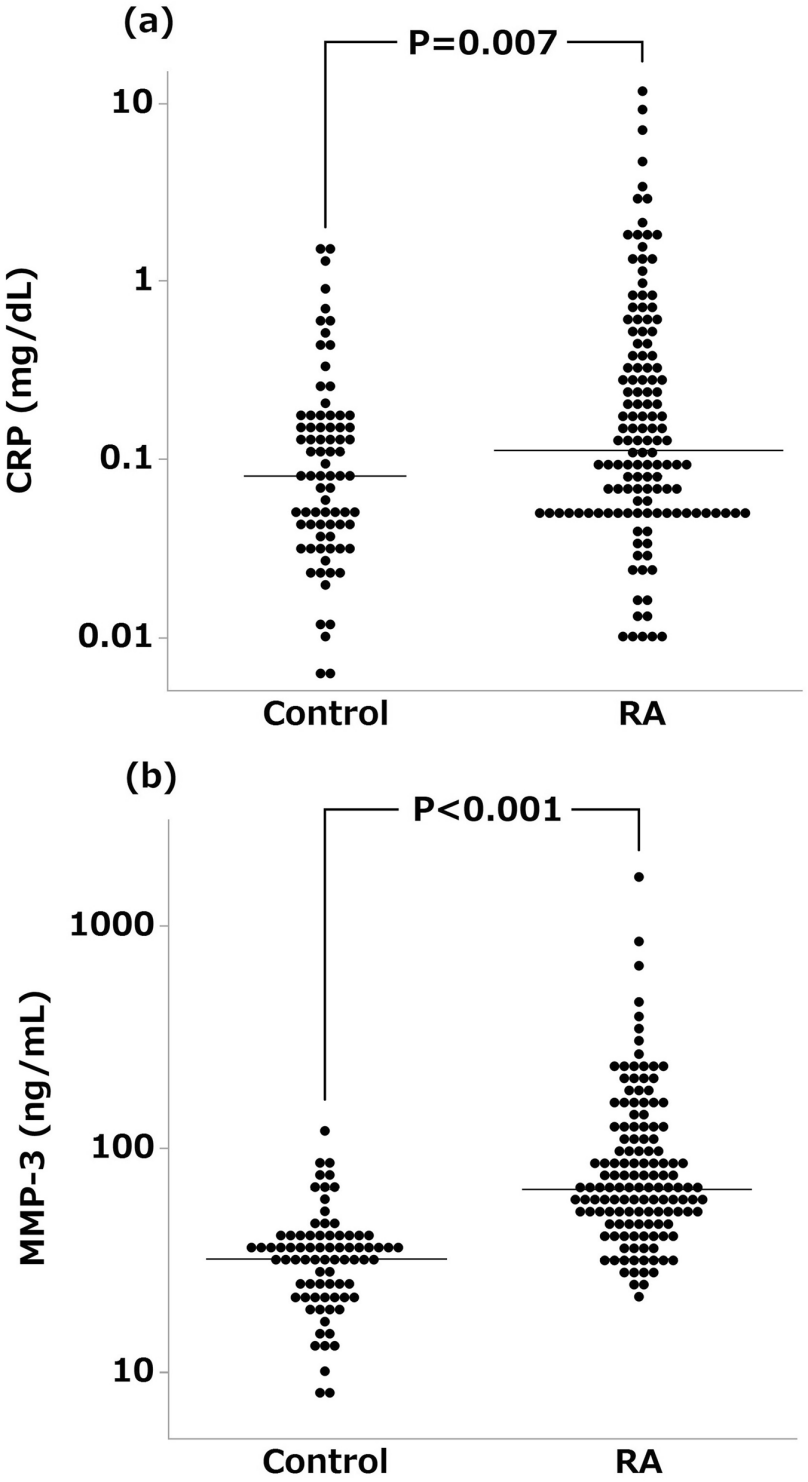
Comparison of serum CRP and MMP-3 concentrations between patients with RA and controls. Serum concentrations of C-reactive protein (CRP) (**a**) and matrix metalloproteinase-3 (MMP-3) (**b**) in 136 patients with RA and 78 controls are shown. The horizontal line indicates the median value in each group. Wilcoxon rank sum test was used for the statistical analyses. *P* < 0.05 was considered statistically significant. Note that the vertical line indicates logarithmic scale.

Then, we examined the relationships of serum concentrations of leptin and adiponectin with inflammatory markers and clinical data in patients with RA. CRP and MMP-3 are routinely used major biomarkers to follow the activity of RA. Figure 3 shows serum concentrations of CRP and MMP-3 in patients with RA and controls. Serum concentration of CRP in patients with RA (median 0.11 mg/dL, IQR 0.05–0.36 mg/dL) was significantly higher than that in controls (0.08 mg/dL, 0.04–0.15 mg/dL). Serum MMP-3 concentration was also significantly higher in patients with RA (66.1 ng/mL, 47.8–122.6 ng/mL) than that in controls (31.8 ng/mL, 22.8–38.8 ng/mL).

Table 1 shows the results of multiple regression analysis of serum concentrations of leptin and adiponectin with serum levels of inflammatory markers, such as CRP and MMP-3, and clinical data, such as Steinbrocker stage, ACR stage, and disease duration in patients with RA. Correlations of serum leptin concentrations with inflammatory markers and clinical data varied when serum leptin concentrations were expressed in different ways. Non-normalized serum leptin concentration had a significant positive and negative correlations with serum CRP and MMP-3 concentrations, respectively. Serum leptin concentration normalized by BMI had a positive correlation with serum CRP concentration, while that normalized by body fat mass had a positive correlation with ACR clinical stage. Serum adiponectin concentrations, expressed in any ways, showed a significant positive correlation with Steinbrocker stage. In addition, serum adiponectin concentration normalized by body fat mass had a significant positive correlation with serum MMP-3 concentration. In controls, serum leptin and adiponectin concentrations expressed in any ways did not have significant correlations with these inflammatory markers as shown in Table 2.

**Table 1:** Multiple regression analysis of serum concentrations of leptin and adiponectin with serum levels of inflammatory markers, as well as clinical data, in patients with RA.

Inflammatory markers and clinical data	Leptin			Leptin/BMI^1^			Leptin/Fat kg^2^		
	β^3^	t^4^	P^5^	β	t	P	β	t	P
CRP	0.30	2.8	0.006	0.29	2.7	0.008	0.11	1.1	0.292
MMP-3	− 0.23	− 2.1	0.036	− 0.19	− 1.8	0.080	0.11	1.0	0.317
Steinbrocker stage	− 0.06	− 0.6	0.549	− 0.09	− 0.9	0.371	− 0.13	− 1.3	0.185
ACR clinical stage	0.09	1.0	0.344	0.11	1.1	0.269	0.22	2.3	0.022
Disease duration	− 0.003	− 0.03	0.978	0.01	0.1	0.890	0.04	0.4	0.685

Inflammatory markers and clinical data	Adiponectin			Adiponectin/BMI^1^			Adiponectin/Fat kg^2^		
	β	t	P	β	t	P	β	t	P
CRP	− 0.11	− 1.1	0.280	− 0.13	− 1.2	0.216	− 0.17	− 1.7	0.102
MMP-3	0.10	0.9	0.369	0.11	1.0	0.301	0.24	2.3	0.024
Steinbrocker stage	0.27	2.8	0.006	0.25	2.6	0.010	0.19	2.0	0.049
ACR clinical stage	− 0.10	− 1.0	0.311	− 0.10	− 1.1	0.296	− 0.005	− 0.1	0.961
Disease duration	0.10	1.1	0.286	0.08	0.9	0.368	0.10	1.1	0.288

*ACR* American College of Rheumatology, *BMI* Body Mass Index, *CRP* C-reactive protein, *MMP-3* matrix metalloproteinase-3.

^1^Serum leptin and adiponectin concentrations corrected for (divided by) BMI.

^2^Serum leptin and adiponectin concentrations corrected for (divided by) body fat kg.

^3^β: standardized partial regression coefficient.

^4^| t |≥ 2 is considered to be statistically significant.

^5^*P* < 0.05 is statistically significant.

**Table 2:** Multiple regression analysis of serum concentrations of leptin and adiponectin with serum levels of CRP and MMP-3 in controls.

Inflammatory markers and clinical data	Leptin			Leptin/BMI			Leptin/Fat kg		
	β	t	P	β	t	P	β	t	P
CRP	0.03	0.24	0.814	0.02	0.16	0.875	0.04	0.39	0.701
MMP-3	− 0.10	− 0.88	0.381	− 0.12	− 1.04	0.300	− 0.06	− 0.48	0.631

Inflammatory markers and clinical data	Adiponectin			Adiponectin/BMI			Adiponectin/Fat kg		
	β	t	P	β	t	P	β	t	P
CRP	− 0.22	− 1.92	0.059	− 0.22	− 1.97	0.053	− 0.20	− 1.79	0.078
MMP-3	− 0.10	− 0.88	0.380	− 0.08	− 0.73	0.466	− 0.06	− 0.53	0.595

### Serum leptin and adiponectin concentrations in RA patients with or without biological DMARDs treatment

Patients with RA treated with biological DMARDs (n = 48) had a significantly lower serum CRP concentration [0.05 mg/dL (0.04–0.13 mg/dL)] than that not receiving biological DMARDs [n = 88; 0.16 mg/dL (0.08–0.55 mg/dL)] (*P* < 0.001). Serum leptin concentrations expressed in any ways were significantly lower in patients treated with biological DMARDs than those not receiving them [14.2 ng/mL (7.5–23.6 ng/mL) vs. 20.7 ng/mL (12.7–32.9 ng/mL), *P* = 0.001; 0.67 ng/mL/BMI (0.38–1.05 ng/mL/BMI) vs. 0.93 ng/mL/BMI (0.58–1.42 ng/mL/BMI), *P* < 0.001; 0.92 ng/mL/kg fat (0.59–1.55 ng/mL/kg fat) vs. 1.29 ng/mL/kg fat (0.96–1.94 ng/mL/kg fat), *P* = 0.001]. Serum adiponectin concentrations expressed in any ways were not significantly different between patients treated with biological DMARDs and those not receiving them [12.1 µg/mL (7.8–16.6 µg/mL) vs. 10.6 µg/mL (6.6–16.1 µg/mL), *P* = 0.261; 0.59 µg/mL/BMI (0.34–0.85 µg/mL/BMI) vs. 0.49 µg/mL/BMI (0.29–0.74 µg/mL/BMI), *P* = 0.232; 0.93 µg/mL/kg fat (0.42–1.62 µg/mL/kg fat) vs. 0.71 µg/mL/kg fat (0.39–1.48 µg/mL/kg fat), *P* = 0.213]. Serum MMP-3 concentration, Steinbrocker stage, ACR clinical stage and disease duration were not significantly different between patients treated with biological DMARDs and those not receiving them. The other anti-rheumatic drugs did not show any significant influence on serum leptin and adiponectin concentrations.

## Discussion

The current study demonstrated that serum concentrations of leptin and adiponectin were positively and negatively correlated with body fat mass, respectively. This finding is in agreement with previous reports of high serum leptin levels and low serum adiponectin levels in obese patients^1,2,14^. Because both leptin and adiponectin are primarily produced in adipose tissue, it is not surprising that serum leptin concentrations correlated positively with the amount of body fat. In addition, obesity could result from insensitivity to leptin and such resistance would increase circulating leptin^1^. The negative correlation between adiponectin and body fat mass could be explained by the effects of TNF-α. Obesity is a chronic inflammatory condition, in which TNF-α is overproduced^17^, and TNF-α has been shown to suppress the transcription of adiponectin in an adipocyte cell line^3^.

The majority of previous studies reported that serum leptin concentrations are increased in RA^9,10^, whereas a few reported no differences^18^. For adiponectin, most previous studies reported increased serum adiponectin concentrations in RA^11,12^, whereas some reported decreased^19^ or unchanged^20^. One explanation for such discrepancies may be the influence of body fat mass on adipocytokines. We re-evaluated serum leptin and adiponectin concentrations using non-normalized data, normalized ones by BMI and normalized ones by body fat mass and confirmed that serum leptin and adiponectin concentrations are elevated in RA.

Several reports have indicated that leptin is involved in inflammatory processes and the immune system^3^. CRP reflects the intensity of systemic inflammation and tissue destruction^21^. In the pathogenesis of RA, joint synovial membranes are a major site of inflammation where cytokines such as TNF-α, IL-1, and IL-6 are produced^6^. TNF-α has been reported to stimulate leptin secretion from human adipose tissues^22^, and leptin stimulates TNF-α secretion from human macrophages^23^ in adipose tissue. MMP-3 is a good indicator of synovitis in joints^24^, and leptin has been reported to enhance MMP-3 production in vitro in cartilage tissues from patients with osteoarthritis^25^. Multiple regression analysis revealed that non-normalized serum leptin concentration correlated positively with CRP and negatively with MMP-3, BMI normalized one with CRP and body fat mass normalized one with ACR clinical stage. Patients with RA receiving biological DMARDs had lower CRP concentrations as well as lower leptin concentrations expressed in any ways than those without receiving them. Although we could not find any significant correlation between leptin concentration normalized by body fat mass and inflammatory markers such as CRP and MMP-3, the latter finding may support previous reports showing the pro-inflammatory nature of leptin^7^. Whether leptin is associated with the process of erosion of the joints or not has long been debated; protective, deteriorating or no effects^7,10^. The present finding that serum leptin did not have any correlation with Steinbrocker stage suggests that leptin may not be involved in the process of erosion of the joints in RA.

Adiponectin reportedly plays important roles in inflammatory processes and the immune system^5,11,13^. Adiponectin exhibits anti-inflammatory properties such as suppressing the synthesis of pro-inflammatory cytokines such as TNF-a and IL-6. On the other hand, adiponectin reportedly stimulates MMP-3 production in joints and promotes local inflammation and tissue destruction^12^. Thus, adiponectin seems to play controversial roles in RA, that is, anti-inflammatory effects and pro-inflammatory effects in different situations^12^. The present study demonstrated that adiponectin concentrations expressed in any ways correlated positively with Steinbrocker stage and adiponectin concentration normalized by body fat mass correlated positively with MMP-3 concentration as well. These findings suggest that adiponectin may exert local pro-inflammatory effects via MMP-3 and contribute to bone and cartilage destruction in RA.

Because this is cross-sectional research in a clinical setting, many factors are involved leading to a not so remarkable difference and correlation among groups even though it is statistically significant. This is a limitation of this study but we believe that our results might have shed some light on the involvement of leptin and adiponectin in the pathogenesis of RA.

## Conclusions

Serum leptin and adiponectin concentrations normalized by body fat mass were elevated in RA. Adiponectin but not leptin may be involved in joint damage in RA.

## Methods

### Participants

We examined 136 patients with RA (26 males and 110 females, 69.6 ± 9.3 years) and 78 controls without RA (36 males and 42 females, 66.7 ± 15.0 years). Patients with RA were those who were attending to the outpatient clinic at our hospitals without any serious co-morbidities. Controls without RA included patients who had orthopedic problems such as joint pains due to osteoarthritis or osteoporosis. Those who gave written informed consent between December 2018 and August 2020 participated in this study. Clinical characteristics of both groups are shown in Table 3. For patients diagnosed before 2010, RA diagnosis was made using the 1987 ACR (American College of Rheumatology) diagnostic criteria^26^. After 2010, diagnosis was made according to the ACR/EULAR (European League Against Rheumatism) diagnostic criteria^27^. The Steinbrocker classification system^28^ was used to evaluate the degree of bone and cartilage destruction observed on joint X-rays: stage 1, no destruction; stage 2, thinned cartilage and a narrowed joint space but no bone destruction; stage 3, bone or cartilage destruction; and stage 4, joint destruction with ankylosis. The ACR global functional status in RA system^29^ was used to classify the degree of impairment of daily activities: stage 1, completely able to perform usual activities of daily living (self-care, vocational, and avocational); stage 2, able to perform usual self-care and vocational activities but limitations during avocational activities; stage 3, able to perform usual self-care activities but limitations during both vocational and avocational activities; and stage 4, limited ability to perform usual self-care, vocational, and avocational activities. Patients were taking various biological DMARDs, including etanercept (n = 18), tocilizumab (n = 12), golimumab (n = 8), infliximab (n = 3), abatacept (n = 3), adalimumab (n = 2), and certolizumab (n = 2), as well as other anti-rheumatic drugs, such as methotrexate (n = 99), prednisolone (n = 48), sulfasarazine (n = 34), bucillamine (n = 26), iguratimod (n = 13), and tacrolimus (n = 11). One patient with type 2 diabetes mellitus was excluded from this study because his serum leptin concentration normalized by body fat mass was judged as an outliner by the Smirnov test. Blood was obtained in the morning, the serum was immediately separated and the aliquots of the serum samples were stored at –30˚C for no more than two years until the assays. Repeated freeze thaw cycles were avoided to prevent sample degradation.

**Table 3:** Clinical characteristics of subjects.

Characteristic	Controls	RA	*P* value
No. of subjects (male/female)	78 (36/42)	136 (26/110)	
Age (years)	66.7 ± 15.0	69.6 ± 9.3	0.079
Disease duration (months)	ND	134.0 ± 111.7	
Height (cm)	160.7 ± 8.8	155.1 ± 8.9	< 0.001
Weight (kg)	61.1 ± 12.8	54.4 ± 13.4	< 0.001
BMI (kg/m^2^)	23.5 ± 3.6	22.4 ± 4.0	0.054
Body fat mass (kg)	17.6 ± 5.0	16.0 ± 7.6	0.096
Body muscle mass (kg)	10.7 ± 2.3	12.2 ± 3.1	0.016
Body bone mass (kg)	2.2 ± 0.4	2.1 ± 0.5	0.404
**Steinbrocker stage**^**1**^	ND	2.8 ± 1.0	
Stage 1		17	
Stage 2		31	
Stage 3		53	
Stage 4		35	
**ACR stage**^**2**^	ND	1.9 ± 0.7	
Stage 1		39	
Stage 2		75	
Stage 3		22	
Stage 4		0	
Leptin (ng/mL)	13.8 (8.7–23.3)	18.9 (10.9–28.2)	0.021
Adiponectin (µg/mL)	7.7 (5.7–11.1)	11.5 (7.0–16.2)	< 0.001

Data are mean ± standard deviation or number.

*ACR* American College of Rheumatology, *BMI* body mass index, *ND* not determined, *RA* rheumatoid arthritis.

Wilcoxon rank sum test was used to compare leptin and adiponectin concentrations between groups.

^1^Steinbrocker stage is based on changes in joint X-ray images.

^2^ACR stage is based on the degree of impairment of daily life.

Student’s *t* test was used to compare the characteristics between groups, with *P* < 0.05 considered statistically significant.

### Analysis of body composition

The body height and weight were measured using scales. Body composition, including fat, muscle and bone masses, was measured using a dual-frequency body composition Analyzer DC-430A (Tanita Japan Co. Ltd., Tokyo, Japan). This instrument performs tetra-polar foot-to-foot bioelectrical impedance analysis, which measures the electric resistance by applying a weak electrical current to the distal part of each foot and measuring the voltage at the proximal part of each foot. This system utilizes multi-frequency measurement and reactance technology to generate accurate data. As the algorithm was based on a large quantity of data, the body composition measured by this system is highly correlated with measurements obtained by dual energy X-ray absorptiometry^30^.

### Enzyme immunoassay of leptin, adiponectin, C-reactive protein and matrix metalloproteinase 3

Enzyme immunoassay (EIA) of leptin followed our previously described method^31^. In brief, human leptin antibody was generated in rabbits by multiple subcutaneous injections of human leptin (AFP496C; National Hormone and Peptide Program [NHPP], Harbour-UCLA Medical Center, Torrance, CA, USA). Fab’-horseradish peroxidase conjugate was made using maleimide method and then used as a detection antibody. Another human anti-leptin antibody (AFP6621299; NHPP) was adsorbed to polystyrene balls (Precision Plastic Ball Co., Chicago, IL, USA) and used as a capture antibody. Human leptin (AFP496C, NHPP) was used for the reference preparation. The assay range was 0.01–10 ng/mL, and the intra- and inter-assay coefficients of variation were 6.5% and 9.8%, respectively. EIA for adiponectin was performed in a similar manner, using anti-human adiponectin antibodies (10-7816 as the detection antibody and 10-7815 as the capture antibody) purchased from Fitzgerald Industries International (Acton, MA, USA). Human adiponectin protein (30–1800; Fitzgerald Industries International) was used for the reference preparation. The assay range was 0.01–10 ng/mL, and the intra- and inter-assay coefficients of variation were 5.5% and 7.8%, respectively. Serum leptin and adiponectin concentrations were expressed in three ways: raw data, those normalized by BMI (raw data divided by BMI) and those normalized by body fat mass [raw data divided by the amount of fat (kg)]. Human C-reactive protein (CRP) and human matrix metalloproteinase 3 (MMP-3) were measured using the Quantikine ELISA Kit for CRP and MMP-3, respectively (R & D systems, Minneapolis, MN, USA). All serum samples were diluted 10–5000 times so that the concentrations of leptin, adiponectin, CRP and MMP-3 entered their assay ranges. They were all measured in duplicate.

## Statistics

Normally distributed data were expressed as mean ± standard deviation (SD) and analyzed using Student’s *t* test. Non-normally distributed data were expressed as median (interquartile range [IQR]) and compared using the Wilcoxon rank sum test. Pearson correlation analysis was used to examine the relationship between serum concentrations of leptin and adiponectin and body fat mass. Multiple regression analysis was used to examine the relationship of serum leptin and adiponectin concentrations with serum levels of inflammatory markers, as well as clinical data. Statistical analysis was performed using JMP version 14 software (© SAS Institute Inc., Tokyo, Japan). *P* < 0.05 was considered statistically significant.

### Ethics approval and consent to participate

This study was approved by the Ethics Review Committees of Kansai Medical University Hospital, Takarazuka Hospital, Miyashima Rheumatism Orthopedic Clinic, and Sugano Orthopedic Clinic. Informed consent was obtained from all participants. This study was carried out in accordance of the Declaration of Helsinki.

## Data Availability

The datasets used and analyzed during the current study are available from the corresponding author on reasonable request.
